# Isolation of C_11_ Compounds and a Cyclopropane Fatty Acid from an Okinawan Ascidian, *Diplosoma* sp.

**DOI:** 10.3390/molecules16129972

**Published:** 2011-12-02

**Authors:** Tamanna Rob, Takayuki Ogi, Wilmar Maarisit, Junsei Taira, Katsuhiro Ueda

**Affiliations:** 1 Department of Chemistry, Biology and Marine Science, University of the Ryukyus, Nishihara-cho Okinawa 903-0123, Japan; Email: ogitkyuk@pref.okinawa.lg.jp (T.O.); 2 Okinawa Industrial Technology Center, Uruma-shi Okinawa 904-2234, Japan; 3 Department of Bioresources Engineering, Okinawa National College of Technology, Nago-shi Okinawa 905-2192, Japan; Email: taira@okinawa-ct.ac.jp (J.T.)

**Keywords:** ascidian, *Diplosoma* sp., cytotoxity, NMR

## Abstract

Pentylphenols **1** and **2**, cyclopropane fatty acid **3**, and cyclopentenones **4** and **5**, were isolated from an ascidian, *Diplosoma* sp. The structures of **1**−**5** were determined by spectroscopic analysis and/or synthesis. Compound **1** inhibited the division of fertilized sea urchin eggs and compound **4** showed mild cytotoxity against HCT116 cells (human colorectal cancer cell).

## 1. Introduction

**Figure 1:**
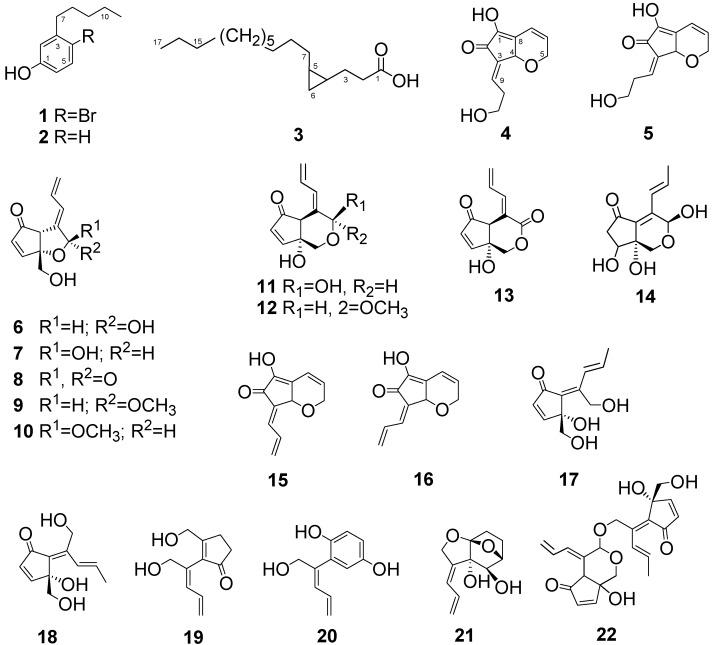
C_11_ compounds and a cyclopropane fatty acid from marine organisms.

Ascidians are a rich source of novel bioactive secondary metabolites, including a diverse array of amino acid-derived alkaloids, cyclic peptides, and acetogenins [1,2,3,4]. The biomedical potential of ascidian metabolites has resulted in a focused interest in these primitive chordates. A series of C_11_ compounds having the distinctive exo-allylidene-lactone named didemnenones were isolated from didemnid ascidians, *Trididemnum cyanophorum* [didemnenones A (**6**) and B (**7**)] and *Didemnum voeltzkowi* (didemnenones C and D) [5]. They showed a wide range of biological activities, including toxicity against leukemia cells as well as antimicrobial and antifungal activities. Their structures were determined based on an X-ray investigation of the corresponding methylacetal and from synthetic results [5,6,7]. As described previously, as part of our ongoing research aimed at the isolation of biologically active metabolites from marine organisms living in the tidal zone, we have isolated fourteen C_11_ compounds, dinemnenone congeners **6**–**18** and **22** from the didemnid ascidians *Lissoclinum* sp. and *Diplosoma* spp. [8,9,10] (Figure 1).

As part of our continuing chemical studies of Okinawan marine organisms, we examined the constituents of the ascidian *Diplosoma* sp. A crude ethyl acetate (EtOAc) extract of this organism strongly inhibited cell division of fertilized sea urchin eggs [11]. Bioassay-guided fractionation of the extract, prepared from the first collection of the ascidian in April, 2003, led to the isolation of a new compound, 4-bromo-3-pentylphenol (**1**) [12], the known 3-pentylphenol (**2**) [12,13], and a new cyclopropane fatty acid **3**. In addition, rapid fractionation of the extract made from a second collection in April 2006 gave new unstable C_11_ compounds **4**[12] and **5**.

## 2. Results and Discussion

The brown, encrusting ascidian *Diplosoma* sp. was collected by hand from the coast of Hateruma Island, Okinawa, and stored at −15 °C before being extracted with acetone. The acetone extract was partitioned between EtOAc and water. The EtOAc extract from the first collection of the ascidian completely inhibited the first cleavage of fertilized sea urchin eggs at 20 ppm. Bioassay-guided fractionation of the toxic extract by a series of chromatographic processes, including silica gel column chromatography (CC), high performance thin layer chromatography (HPTLC) and high performance liquid chromatography (HPLC), yielded compounds **1** (0.00010%), **2** (0.000050%), and cyclopropane fatty acid **3** (0.00020%). The sample from the second collection was immediately extracted with acetone after transportation to our laboratory, and the extract was partitioned between water and ethyl acetate (EtOAc). The EtOAc extract was suspended in MeOH/H_2_O (1:1) and successively extracted with hexanes and CHCl_3_. The CHCl_3_ extract was quickly separated by HPLC on ODS to yield unstable compounds **4** (0.37%) and **5** (0.057%).

LREIMS of **1** showed the M^+^ ion at *m*/*z* 242.0 and an ion of equal intensity at *m*/*z* 244.0, indicating the presence of a single bromine atom. Analysis of **1** by ^13^C-NMR (Table 1) and LREIMS provided the molecular formula C_11_H_15_BrO, which accounted for four degrees of unsaturation.The presence of a 1,3,5-trisubstituted benzene ring was deduced from ^1^H-NMR and ^13^C-NMR data [δ_H_ 6.07 (dd), δ_C_ 114.8 (d); δ_C_ 114.8 (s); δ_H_ 6.32 (d), δ_C_ 117.5 (d); δ_H_ 7.21 (d), δ_C_ 133.6 (d); δ_C_ 143.5 (s); δ_C_ 155.6 (s)].

**Table 1:** ^1^H- and ^13^C-NMR data for compounds **1**, **4** and **5**.

C no.	1 ^a^				4				5	
	*δ*_C_		*δ*_H_ (mult, *J* in Hz)		*δ*_C_^b^		*δ*_H_ (mult, *J* in Hz) ^b^		*δ*_C_^c^	*δ*_H_ (mult, *J* in Hz) ^b^
1	155.6				147.7				147.4	
2	117.5		6.32 (1H, d, 3.0)		188.0				188.8	
3	143.5				135.5				135.5	
4	114.8				70.8		4.88 (br s)		71.8	4.63 (br s)
5	133.6		7.21 (1H, d, 8.5)		66.9		4.40 (1H, ddd, 2.5, 2.5, 18.5)		67.0	4.37 (1H, ddd, 2.5, 4.5, 18.5)
							4.52 (1H, ddd, 1.6, 4.5, 18.5)			4.48 (1H, ddd, 1.6, 4.5, 18.5)
6	114.8		6.07 (1H, dd, 3.0, 8.5)		133.1		6.07 (1H, ddd, 2.5, 4.5, 10.0)		134.2	6.02 (1H, ddd, 2.5, 4.5, 10.0)
7	36.4		2.57 (2H, t, 8.0)		118.5		6.73 (1H, ddd, 1.6, 2.5, 10.0)		118.3	6.70 (1H, ddd, 1.6, 2.5, 10.0)
8	29.8		1.50 (2H, quin., 8.0)		130.5				128.8	
9	31.8		1.21 (2H, m)		134.7		6.61 (1H, dt, 1.6, 8.8)		134.7	6.22 (1H, dt, 1.6, 8.8)
10	22.8		1.21 (2H, m)		32.1		2.60 (1H, m)		31.0	2.90 (1H, m)
							2.70 (1H, m)			2.90 (1H, m)
11	14.2		0.83 (3H, t, 7.3)		60.5		3.70 (2H, m))		61.9	3.65 (2H, m))
OH			3.96 (1H, br s)							

^a^ ^1^H-NMR (500 MHz) and ^13^C-NMR (125 MHz) recorded in C_6_D_6_; ^b^ ^1^H-NMR (500 MHz) or ^13^C-NMR (100 MHz) recorded in 5% CD_3_OD in CDCl_3_. ^c^ ^13^C-NMR (100 MHz) recorded in CDCl_3_.

**Figure 2:**
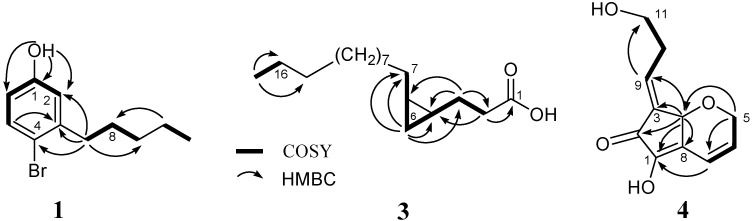
Structures of compounds **1**, **3** and **4** based on 2D NMR data.

**Scheme 1:**
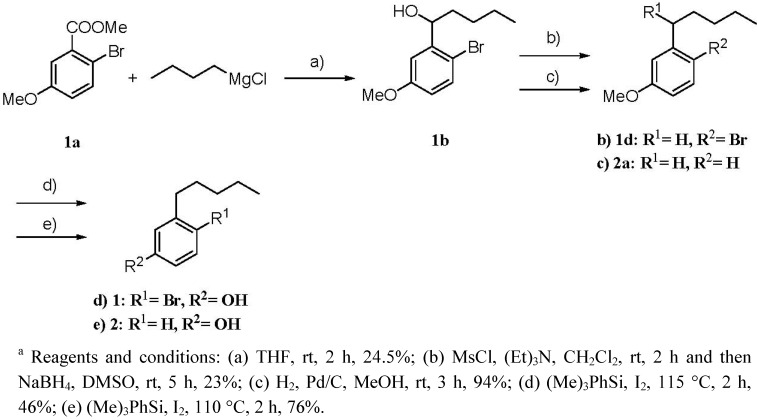
Synthesis of compounds **1** and **2**^a^.

The ^1^H- and ^13^C-NMR spectra also contained five highfield signals [δ_C_ 14.2 (q), δ_H_ 0.83 (3H, t, *J* = 7.3 Hz); δ_C_ 22.8 (t), δ_H_ 1.21 (2H, m); δ_C_ 29.8 (t), δ_H_ (2H, quin., *J* = 8.0 Hz); δ_C_ 31.8 (t), δ_H_ 1.21 (2H, m); δ_C_ 36.4 (t), δ_H_ (2H, t, *J* = 8.0 Hz)]. The methyl protons at δ_H_ 0.83 were coupled to the methylene protons at δ_H_ 1.21. The methylene protons at δ_H_ 2.57 were coupled to the methylene protons at δ_H_ 1.50, which were in turn coupled to the other methylene protons at δ_H_ 1.21 (Table 1, Figure 2). Further detailed interpretation of the ^1^H-NMR, ^13^C-NMR, ^1^H−^1^H COSY and HMQC spectral data revealed the presence of an *n*-pentyl moiety in **1** (Figure 2). The proton at δ_H_ 3.96 (s) did not show any HMQC correlations, but HMBC correlations to two olefinic carbons, suggesting thepresence of an OH group coupled with the molecular formula of **1**. Bromo, *n*-pentyl and hydroxyl positions on the benzene ring were determined by comparison with calculated ^1^H and ^13^C chemical shift values, and by HMBC correlations of OH/C-1, OH/C-2, H-2/C-1, H-2/C-7, H-5/C-3, H-5/C-4, H-5/C-6, H-7/C-2, H-7/C-3, H-7/C-4. Since decomposition of **1** prevented us from characterizing this compound completely, synthesis of **1** was attempted (Scheme 1). The synthesis started with methyl 2-bromo-5-methoxybenzoate (**1a**). The Grignard reaction of **1a** with pentylmagnesium chloride afforded alcohol **1b**. Mesylation of **1b**, followed by reduction of the mesylate with NaBH_4_, gave 4-bromo-3-pentylanisole (**1d**). Cleavage of the ether **1d** with phenyltrimethylsilane/iodine yielded **1** as the sole product. ^1^H-NMR data of the product were identical with those of the naturally occurring compound. The known 3-pentylphenol (**2**) was identified by comparison of its NMR data with those of synthetic **2** (Scheme 1) [12]. Reduction of **2b** with H_2_/Pd-C gave 3-pentylanisole (**2e**). Cleavage of the ether **2e** with phenyltrimethylsilane/iodine yielded **2** as the sole product. ^1^H-NMR data of the product were in agreement with those of natural compound **2**. Compound **1** completely inhibited the first cleavage of fertilized sea urchin eggs at 1 ppm.

Analysis of the ^13^C-NMR and HRFABMS data [*m/z* 269.2462 (M + H)^+^, Δ −1.9 mmu] for compound **3** provided the molecular formula C_1__7_H_32_O_3_, which accounted for two degrees of unsaturation. In the ^13^C-NMR spectrum, 17 carbon signals were observed, including a carbonyl carbon, two methine carbons, 13 methylene carbons and a methyl carbon. The ^13^C-NMR resonance at δ_C_ 176.9 indicated the presence of a carboxyl group, which was substantiated by IR absorption bands at 1700 and 3300 cm^−1^. Four upfield shifted signals [δ_H_ 0.71 (1H, m, H-4), δ_C_ 15.1 (d); 0.71 (1H, m, H-5), δ_C_ 15.9 (d); 0.60 (1H, ddd, *J* = 8.0, 8.0, 5.0 Hz, H-6a) and −0.27 (1H, ddd, *J* = 5.0, 5.0, 5.0 Hz, H-6b), δ_C_ 10.7 (t)] in the NMR spectra suggested that **3** should contain a cyclopropane ring. These data revealed compound **3** to be a cyclopropane fatty acid. Analysis of COSY, HMQC and HMBC spectra permitted assignment of the protons of the cyclopropane ring (Figure 2). Geometric configuration of the cyclopropane was assigned to be *cis* by analysis of the coupling constants (*J*_4, 6a_ = 8.0 Hz, *J*_4, 6b_= 5.0 Hz, *J*_5, 6a_= 8.0 Hz, *J*_5, 6b_= 5.0 Hz, *J*_6a, 6b_= 5.0 Hz). This was confirmed by comparison of the ^1^H- and ^13^C-NMR chemical shifts within the cyclopropane ring for **3**with those of *cis*- and *trans*-1,2-disubstituted cyclopropanes [14,15,16]. Compound **3** showed no activity at 1, 5 and 10 ppm in sea urchin eggs assay.

**Figure 3:**
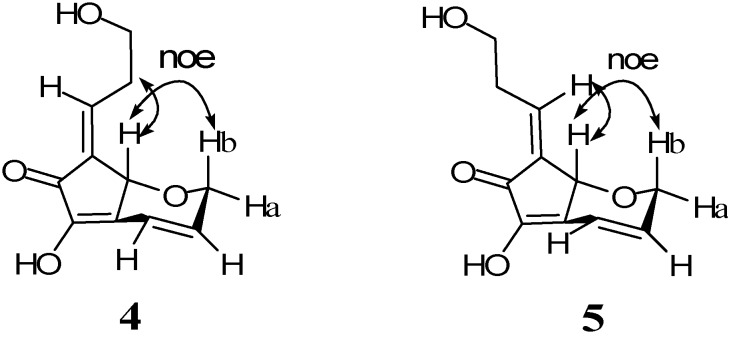
Selected NOEs of compounds **4** and **5**.

Analysis of ^13^C-NMR (Table 1) and HRESIMS data [*m/z* 231.0698 (M + Na)^+^, Δ −6.4 mmu; *m/z* 191.0688 (M + H − H_2_O)^+^, Δ −2.0 mmu] for compound **4** provided the molecular formula C_11_H_12_O_4_, which indicated six degrees of unsaturation. The IR absorption bands at 1,680 and 3,250 cm^−1^ indicated the presence of carbonyl and hydroxyl groups. ^1^H- and ^13^C-NMR data analysis indicated the presence of a carbonyl carbon (δ_C_ 188.0), a *cis* double bond [δ_H_ 6.07 (1H, ddd, *J* = 10.0, 4.5, 2.5 Hz), δ_C_ 133.1 (d); δ_H_ 6.73 (1H, ddd, *J* = 10.0, 2.5, 1.6 Hz ), δ_C_ 118.5 (d)], a tetrasubstituted double bond [δ_C_ 130.5 (s); 147.7(s)], a trisubstituted double bond [δ_H_ 6.61 (1H, td, *J* = 8.8, 1.6 Hz), δ_C_ 134.7 (d); δ_C_ 135.5 (s)], an oxygenated methine [δ_H_ 4.88 (1H, br s), δ_C_ 70.8 (d)], two oxygenated methylenes [δ_H_ 4.40 (1H, ddd, *J* = 18.5, 2.5, 2.5 Hz) and 4.52 (1H, ddd, *J* = 18.5, 4.5, 1.6 Hz), δ_C_ 66.9 (t); δ_H_ 3.70 (2H, m), δ_C_ 60.5 (t)] and a methylene [δ_H_ 2.60 (1H, m) and 2.70 (1H, m), δ_C_ 32.1 (t)]. The NMR data of **4** showed close similarity to those of the known compound **15**[9,17]. However, in contrast to **15**, **4** contained an oxygenated methylene and a methylene instead of a terminal double bond. The oxygenated methylene protons at δ_H_ 4.40 (1H) and 4.52 (1H) were coupled to the olefinic proton at δ_H_ 6.07. The oxygenated methylene protons were also coupled to the olefinic proton at δ_H_ 6.73 with small coupling constants of 2.5 Hz and 1.6 Hz, respectively. The other oxygenated methylene protons at δ_H_ 3.70 were coupled to the methylene protons at δ_H_ 2.60 (1H) and δ_H_ 2.70 (1H), which were in turn coupled to the olefinic proton at δ_H_ 6.61 (Table 1, Figure 2). Further detailed interpretation of the ^1^H-NMR, ^13^C-NMR, ^1^H−^1^H COSY and HMQC spectral data revealed the presence of partial structures, C-5−C-7, C-9−C-11, C-1−C-8, C-3−C-9, C-2 and C-4 (Figure 2). The connectivity of the partial structures was established from the HMBC correlations of H-4/C-1, H-4/C-2, H-4/C-3, H-4/C-8, H-4/C-9, H-5/C-4, H-5/C-7, H-7/C-1, H-9/C-11, as shown in Figure 2, to describe the entire carbon framework of **4**. The double bond stereochemistry of compound **4** was established by NOE Differential Spectroscopy (NOEDS) experiments (Figure 3). Irradiation of H-4 resulted in enhancement of H-10 and H-5b, thereby supporting the *E*-configuration of the double bond between C-3/C-9. The proton H-9 shifts down field at δ_H_ 6.61. Irradiation of H-9 showed no significant enhancement on H-4, and a very small enhancement with H-11. The absolute configuration of C-4 is yet to be determined.

Analysis of ^13^C-NMR (Table 1) and HREIMS data [*m/z* 191.0736 (M + H − H_2_O)^+^, Δ −2.8 mmu] for compound **5** provided the molecular formula C_11_H_12_O_4._ Spectroscopic data for **5** showed close similarity to those of **4** (Table 1). The largest difference in chemical shifts observed between **4** and **5** were for H-9. The chemical shift (δ_H_ 6.61) of H-9 in **4** was at lower field than that (δ_H_ 6.38) in **5** owing to the magnetic anisotropy effect of the carbonyl group, suggesting an *E* configuration for the C-3,9 double bond of **4** and thus a *Z* configuration for that of **5**. This was confirmed by NOEDS experiments. Irradiation of H-4 of **4** resulted in enhancement of H-9 and H-5b, and H-4 of **5** showed NOEs to H-10 and H-5b. Compound **4** showed weak cytotoxity against HCT116 cells (human colorectal cancer cells) in a dose dependent manner (IC_50_: >20 ppm). We could not evaluate the activity of unstable compound **5** because of the loss of **5** with the formation of insoluble material.

To date, a variety of C_11_ compounds **6**–**21**have been isolated from ascidians, sponges and cyanobacteria [5,9,10,17,18,19,20]. C_11_ cyclopentenones (didemnenones) **13**, **14** and **19**, the related compound **20**, and compound **21**, have been isolated from ascidians (*Lissoclinum* spp.), cyanobacteria, and a sponge, respectively. Compound **18** has been isolated from an ascidian (*Diplosoma virens*) and a sponge (*Ulosa* sp.) [9,17]. Isolation of a series of C_11_ compounds, including compounds **4** and **5**, from unrelated marine organisms supports the potential microbial origin of these compounds. From this perspective, it was assumed that the ascidian *Diplosoma* sp. might not be the real producer of compounds **4** and **5**; rather, these compounds could originate from a microbial source such as a *Prochloron* sp., an obligatory symbiont of ascidians [21,22,23]. The *Prochloron* sp. was isolated from ascidian *Diplosoma* sp. by squeezing through a plankton net, followed by acetone extraction. ^1^H-NMR spectra of the crude extract showed the same peaks as those of pure compounds **4** and **5**. Therefore, it was concluded that a microorganism, probably a *Prochloron* sp. was the actual producer of **4** and **5**.

Most C_11_ compounds are derived from polyketides [(six acetates − C_1_) or (five acetates + C_1_)]. Pentylphenols are known to be formed with a loss of CO_2_ from a C_12_ parent (six acetates) [24]. In the previous paper, we proposed that didemnenone-related compounds **6-22** should be derived from 4-methyldecane via various types of cyclization [10,24]. Investigation of the biogenesis of compounds **4** and **5**, and related compounds **6**−**18** is in progress in our laboratory.

## 3. Experimental

### 3.1. General Experimental Procedures

Optical rotations were measured on a JASCO P-1020 polarimeter. UV spectra of the methanol solutions were measured on a JASCO V-550 spectrophotometer. IR spectra were recorded on a JASCO FT/IR-300 spectrometer. The ^1^H-, ^13^C-, and 2D-NMR spectra were recorded on a JEOL lambda 400 or a JEOL α-500 spectrometer, and ^1^H and ^13^C chemical shifts were referenced to the solvent peaks (δ_H_ 7.24 and δ_C_ 77.0 in CDCl_3_; δ_H_ 7.16 and δ_C_ 128.6 in C_6_D_6_). Mass spectra were measured on a Waters Quattro micro API triple quadruple mass analyzer. Open column chromatography was performed on Kieselgel 60 (70–230 mesh, Merck). HPLC was performed using a COSMOSIL-packed ODS HPLC column (C18, 10 × 250 mm) or COSMOSIL Si60 HPLC column (5SL, 10 × 250 mm). Analytical TLC was performed using Kieselgel 60 F_254_ DC-fertigplatten (Merck). All solvents used were reagent grade.

### 3.2. Animal Material

The small brown tunicate was collected at low tide from the coast of Hateruma Island, Okinawa, Japan in April, 2003 and in April, 2006, and identified as *Diplosoma* sp. by Professor Euichi Hirose, University of the Ryukyus, Japan. A voucher specimen was deposited at the University of the Ryukyus (specimen no. 030412).

### 3.3. Extraction and Isolation

The brown tunicate collected from Hateruma Island was kept frozen during transportation. Theascidian *Diplosoma* sp. (1 kg, wet weight) was extracted with acetone (1.5 L) twice. After filtration, the extracts were concentrated *in vacuo* to give an acetone extract. The acetone extract was partitioned between H_2_O (200 mL) and EtOAc (300 mL × 2). The EtOAc extract (21.3 g) completely inhibited the first cell division of fertilized sea urchin eggs at 20 ppm. The extract was first chromatographed on silica gel using hexanes with increasing proportions of EtOAc [hexanes (600 mL) → hexanes/EtOAc (5:1, 600 mL → 3:1, 600 mL → 1:1, 600mL → 3:1, 600 mL) and then EtOAc with increasing proportions of MeOH [EtOAc (600 mL) → EtOAc/MeOH (9:1, 600 mL → 7:1, 600 mL)] to give 12 fractions. An active fifth fraction (240 mg) was subjected to further separation by CC on silica gel using the gradient solvent mixture hexanes-CH_2_Cl_2_-MeOH to give 19 fractions. Active fractions were combined and the mixture (100 mg) was subjected to ODS column chromatography (100% MeOH) to give 14 fractions. The second fraction (10.6 mg) was purified by HPTLC on silica gel using hexanes-EtOAc (6:1) to afford **1** (1.0 mg) and **2** (0.5 mg). The fifth fraction (21 mg) was separated by HPLC on silica gel using hexanes-CHCl_3_-isopropanol (50:10:3) to afford crude **3** (12 mg), which was purified by reversed phased HPLC on ODS using 0.01 M NH_4_Cl in MeCN-MeOH-H_2_O (50:48:2) to give **3** (2.0 mg). The sample of second collection (7 g, wet weight) was soaked in 50 mL acetone at room temperature (rt) and left in the dark for 8 h. After filtration, the residue was again extracted with acetone. The acetone extracts were concentrated under reduced pressure to give a residual oil. The oil was quickly partitioned between H_2_O and EtOAc three times to give 123.2 mg of EtOAc extract. This extract was suspended in MeOH and H_2_O (1:1) and then successively extracted with hexanes and chloroform (CHCl_3_), which were evaporated to give hexanes extract and CHCl_3_ extract. The CHCl_3_ part was subjected to reversed phased HPLC on ODS using MeOH-H_2_O (7:3) to furnish compound **4** (20 mg) in pure form, and a mixture of compound **4** and related compound **5** (10 mg). Further purification of the mixture by HPLC on ODS using MeOH-H_2_O (7:3) to give **4** (6 mg) and **5** (2 mg).

*4-Bromo-3-pentylphenol* (**1**). Colorless oil; ^1^H- and ^13^C-NMR (CDCl_3_) data, see Table 1; LREI(+)MS (relative intensity) *m/z* 244 (M^+^, 36), 242 (M^+^ + 2, 36), 109 (M^+^ − C_4_H_9_ − Br + H, 100).

*cis-3-(2-Undecylcyclopropyl)propionic acid* (**3**). Colorless powder; [*α*]^25^_D_ + 11 (*c* 0.026 CHCl_3_); FT/IR (film) *ν*_max_ 2950, 2830, 1700 cm^−1^; ^1^H-NMR (CDCl_3_, 500 MHz) δ 2.44 (2H, t, *J* = 7.5 Hz, H2), 1.75 (1H, m, H3), 1.49 (1H, m, H3), 1.36 (1H, m, H7), 1.2–1.3 (14H), 1.15 (1H, m, H7), 0.86 (3H, t, *J* = 6.5 Hz, H17), 0.71 (1H, m, H4), 0.71 (1H, m, H5), 0.60 (1H, ddd, *J* = 8.0, 8.0, 5.0 Hz, H6a), −0.27 (1H, ddd, *J* = 5.0, 5.0, 5.0 Hz, H6b); ^13^C-NMR (CDCl_3_, 125 MHz) δ 176.85, 33.98, 31.87, 30.09, 29.65, 29.60, 29.57, 29.30, 29.18, 28.59, 24.10, 22.63, 15.95, 15.08, 14.05, 10.74; HRAPCIMS *m/z* (M + H)^+^ 269.2462 (calcd for C_17_H_32_O_3_, 269.2481).

*(E)-5-Hydroxy-7-(3-hydroxypropylidene)-7,7a-dihydrocyclopenta[b]pyran-6(2H)-one* (**4**). Colorless oil; [*α*]^29^_D_ + 2.4 (*c* 0.060 CHCl_3_); UV (MeOH) *λ*_max_ 227 (log*ε* 4.0), 330 (log*ε* 3.7) nm; FT/IR (film) *ν*_max_ 3350, 2920, 2850, 1685, 1420, 1150, 1046 cm^−1^; ^1^H-NMR (CDCl_3_, 500 MHz) and ^13^C-NMR (CDCl_3_) data, see Table 1; HRESI(+)MS *m/z* (M + Na)^+^ 231.0698 (calcd for C_11_H_1__2_O_4_Na, 231.0634), *m/z* (M + H − H_2_O)^+^ 191.0688 (calcd for C_11_H_11_O_3_, 191.0708).

*(Z)-5-Hydroxy-7-(3-hydroxypropylidene)-7,7a-dihydrocyclopenta[b]pyran-6(2H)-one* (**5**). Colorless oil; [*α*]^25^_D_ + 5.4 (*c* 0.058 CHCl_3_); UV (MeOH) *λ*_max_ 228 (log*ε* 4.0), 330 (log*ε* 3.6) nm; FT/IR (film) *ν*_max_ 3350, 2920, 2850, 1684, 1420, 1151, 1058 cm^−1^; ^1^H- and ^13^C-NMR (CDCl_3_) data, see Table 1; HRESI(+)MS *m/z* (M + H − H_2_O)^+^ 191.0736 (calcd for C_11_H_11_O_3_, 191.0708).

*4-Bromo-3-(1-hydroxybutyl)anisole* (**1b**). To a solution of methyl 2-bromo-5-methoxybenzoate (**1a**) [243 µL (369 mg), 1.51 mmol, Tokyo Kasei Co., Ltd.] in tetrahydrofuran (THF, 2.0 mL) was added butylmagnesium chloride (0.91 M solution in THF, 2.4 mL, 2.12 mmol, Kanto Chemical Co., Inc.) via syringe at rt. The mixture was stirred at rt for 6 h and quenched with saturated NH_4_Cl solution. The products were extracted with CHCl_3_. The CHCl_3_ solution was dried (Na_2_SO_4_) and concentrated *in vacuo*. The residual oil (430 mg) was purified by column chromatography on silica gel (20 g) using hexanes-EtOAc (9:1) to afford alcohol **1b** (101 mg, 24.5%). Colorless solid; mp 42−44 °C; UV (MeOH)_max_ 228 (log*ε* 4.0), 281 (log*ε* 3.2) nm; FT IR *ν*_max_ (KBr) 3300, 3005, 2960, 2920, 2830, 100, 1580, 1460 cm^−1^; ^1^H-NMR (500 MHz, CDCl_3_) δ 7.35 (1H, d, *J* = 8.8 Hz), 7.08 (1H, d, *J* = 3.2 Hz), 6.65 (1H, dd, *J* = 8.8, 3.2 Hz), 4.97 (1H, d, *J* = 8.3, 4.1 Hz), 3.77 (3H, s), 1.72 (1H, m), 1.61 (1H, m), 1.45 (1H, m), 1.35 (1H, m), 0.89 (3H, t, *J* = 7.3 Hz); ^13^C-NMR (125 MHz, CDCl_3_) δ 159.3, 145.0, 133.2, 114.7, 112.5, 112.2, 73.0, 55.5, 37.4, 28.0, 22.5, 14.0; LRESI(+)MS (relative intensity) *m/z* 295 [(M + Na)^+^, 100] and 297 [(M + 2 + Na)^+^, 100].

*4-Bromo-3-butylanisole* (**1d**). To a solution of alcohol **1b** (43 mg, 0.16 mmol) and triethylamine [(183 µL) 132 mg, 1.30 mmol) in dichloromethane (2.0 mL) was added methanesulfonyl chloride [100 µL (148 mg), 1.29 mmol] via syringe at rt. After being stirred at rt for 2 h, the mixture was quenched with methanol (0.1 mL) and stirred at rt for 1 h. The mixture was diluted with H_2_O (5 mL) and the products were extracted with CHCl_3_. The CHCl_3_ solution was dried (Na_2_SO_4_) and concentrated *in vacuo*. The residual oil (75 mg) was then separated by CC [silica gel (500 mg), 100% hexanes] to give crude mesylate **1c** (47 mg). The mesylate (47 mg) was dissolved in dimethyl sulfoxide (DMSO, 2 mL). To the solution was added a solution of NaBH_4_ (46 mg, 1.2 mmol) in DMSO (2 mL) via syringe. The mixture was stirred at rt for 24 h and quenched with 8 drops of acetone. After being stirred for 1 h, H_2_O (10 mL) was added. The products were extracted with hexanes (5 mL × 3). The hexanes solution was dried over Na_2_SO_4_ and concentrated *in vacuo*. The residue (34 mg) was purified by CC on silica gel (500 mg, 100% hexanes) to afford **1d** (9.2 mg, 23%). UV (MeOH)_max_ 228 (log*ε* 3.9), 281 (log*ε* 3.3) nm; FT IR *ν*_max_ (KBr) 3010, 2960, 2930, 2860, 1630, 1560, 1460 cm^−1^; ^1^H-NMR (500 MHz, CDCl_3_) δ 7.37 (1H, d, *J* = 8.5 Hz), 6.74 (1H, d, *J* = 2.2 Hz), 6.59 (1H, dd, *J* = 8.5, 2.2 Hz), 3.76 (3H, s), 2.65 (2H, t, *J* = 8.1 Hz), 1.59 (2H, m), 1.33 (4H, m), 0.89 (3H, t, *J* = 6.6 Hz); ^13^C-NMR (100 MHz, C_6_D_6_) δ 160.1, 144.0, 134.1, 117.3, 115.9, 113.7, 55.4, 37.3, 32.4, 30.6, 23.4, 14.8; LRESI(−)MS (relative intensity) *m/z* 241 [(M − CH_3_)^−^, 100] and 243[(M + 2 − CH_3_)^−^, 100]. 

*4-Bromo-3-pentylphenol* (**1**). A solution of ether **1d **(3.0 mg, 0.012 mmol), phenyltrimethylsilane [50 µL (44 mg), 0.29 mmol] and iodine (10.0 mg, 0.0394 mmol) was heated to 115 °C for 2 h. The mixture was concentrated *in vacuo* and the residual oil was purified by preparative TLC [silica gel, hexanes-EtOAc (9:1)] to give 4-bromo-3-pentylphenol (**1**) (1.3 mg, 46%). Colorless oil; UV (MeOH)_max_ 228 (log*ε* 3.9), 281 (log*ε* 3.3) nm; FT IR *ν*_max_ (KBr) 3300, 2960, 2920, 2840, 1600, 1575, 1460 cm^−^^1^; ^1^H-NMR (500 MHz, CDCl_3_) δ 7.33 (1H, d, *J* = 8.5 Hz), 6.69 (1H, d, *J* = 3.0 Hz), 6.52 (1H, dd, *J* = 8.5, 3.0 Hz), 2.62 (2H, t, *J* = 8.8 Hz), 1.55 (2H, m), 1.32 (4H, m), 0.89 (3H, t, *J* = 6.8 Hz). ^1^H NMR (500 MHz, C_6_D_6_) δ 7.20 (1H, d, *J* = 8.5 Hz), 6.32 (1H, d, *J* = 3.0 Hz), 6.06 (1H, dd, *J* = 8.5, 3.0 Hz), 3.98 (1H, br s), 2.58 (2H, t, *J* = 8.0 Hz), 1.52 (2H, quin., *J* = 8.0 Hz), 1.21 (4H, m), 0.83 (3H, t, *J* = 7.3 Hz); ^1^^3^C-NMR (100 MHz, C_6_D_6_) δ 156.0, 143.9, 134.0, 117.9, 115.3, 115.2, 36.8, 32.1, 30.2, 23.2, 14.5; LRESI(−)MS (relative intensity) *m/z* 241 [(M − H)^−^, 100] and 243 [(M + 2 − H)^−^, 100]. 

*3-Butylanisole* (**2a**). To a solution of alcohol **1b** (9.0 mg, 0.0329 mmol) in methanol (1.0 mL) was added a small amount of 5% palladium on activated carbon. The suspension was stirred at rt under an atmosphere of H_2_ for 8 h, the mixture was filtered through Celite and concentrated *in vacuo.* The filtrate was evaporated and the resulting residue (7.5 mg) was then separated by preparative TLC to give ether **2a** (5.5 mg, 94%). Colorless oil; ^1^H-NMR (500 MHz, CDCl_3_) δ 7.17 (1H, t, *J* = 7.6 Hz), 6.75 (1H, br d, *J* = 7.6 Hz), 6.70 (2H, m), 3.76 (3H, s), 2.56 (2H, t, *J* = 7.6 Hz), 1.59 (2H, m), 1.30 (4H, m), 0.87 (3H, t, *J* = 6.6 Hz).

*3-Pentylphenol* (**2**). A solution of ether **2a** (5.0 mg, 0.028 mmol), phenyltrimethylsilane [100 µL (87 mg), 0.579 mmol] and iodine (15 mg, 0.039 mmol) was heated to 110 °C for 2 h. The mixture was concentrated *in vacuo* and the residual oil was purified by preparative TLC [hexanes-EtOAc (10:1)] to give 4-pentylphenol (**2**) (3.5 mg, 76%). Colorless oil; ^1^H-NMR (500 MHz, CDCl_3_) δ 7.12 (1H, t, *J* = 7.6 Hz), 6.73 (1H, br d, *J* = 7.6 Hz), 6.73 (2H, m), 4.70 (1H, br s), 2.53 (2H, t, *J* = 7.5 Hz), 1.56 (2H, m), 1.29 (4H, m), 0.87 (3H, t, *J* = 6.6 Hz); (500 MHz, C_6_D_6_) δ 7.01 (1H, t, *J* = 8.0 Hz), 6.67 (1H, br d, *J* = 8.0 Hz), 6.43 (1H, br s), 6.40 (1H, br d, *J* = 8.0 Hz), 3.96 (1H, br s), 2.41 (2H, t, *J* =7.8 Hz), 1.49 (2H, quin., *J* = 7.8 Hz), 1.22 (4H, m), 0.83 (3H, t, *J* = 7.3 Hz); ^13^C-NMR (100 MHz, C_6_D_6_) δ 156.8, 145.2, 129.9, 121.2, 116.0, 113.2, 36.5, 32.1, 31.7, 23.2, 14.6.

## 4. Conclusions

We have isolated pentylphenols **1** and **2**, cyclopropane fatty acid **3**, and cyclopentenones **4** and **5** from an ascidian, *Diplosoma* sp. The structures of **1**−**5** were determined by spectroscopic analysis and/or synthesis. Compounds **1**, **3**, **4** and **5** were new. Compound **1** inhibited the division of fertilized sea urchin eggs and compound **4** showed mild cytotoxity against HCT116 cells (human colorectal cancer cell). ^1^H-NMR spectra of the extract of the separated *Prochloron* sp. from the body of the ascidian *Diplosoma* sp. showed the presence of the same peaks as present in those of **4** and **5**, suggesting that *Prochloron* sp. is the actual producers of cyclopentenones.
